# Evaluation of postoperative outcomes of minimally invasive distal pancreatectomy for left-sided pancreatic tumors based on the modified frailty index: a retrospective cohort study

**DOI:** 10.1097/JS9.0000000000000670

**Published:** 2023-08-17

**Authors:** Yejong Park, Dae Wook Hwang, Jae Hoon Lee, Ki Byung Song, Eunsung Jun, Woohyung Lee, Bong Jun Kwak, Song Cheol Kim

**Affiliations:** a Department of Surgery, Division of Hepatobiliary and Pancreatic Surgery; b Department of Convergence Medicine, Asan Institute for Life Sciences, University of Ulsan College of Medicine and Asan Medical Center, Seoul, Republic of Korea

**Keywords:** modified frailty index, minimally invasive distal pancreatectomy

## Abstract

**Background:**

This study compared the postoperative outcomes of minimally invasive distal pancreatectomy (MIDP) for left-sided pancreatic tumors based on the modified frailty index (mFI).

**Materials and methods:**

This retrospective study included 2212 patients who underwent MIDP for left-sided pancreatic tumors between 2005 and 2019. Postoperative outcomes, including complications (morbidity and mortality), were analyzed using mFI, and the participants were divided into two groups: frail (*n*=79) and nonfrail (*n*=2133). A subanalysis of 495 MIDPs for pancreatic ductal adenocarcinoma was conducted to compare oncological outcomes.

**Results:**

Clinically relevant postoperative pancreatic fistula was significantly higher in the frail group than in the nonfrail group. A significant between-group difference was observed in overall complications with Clavien−Dindo classification grade ≥III. Furthermore, the proportion of all complications before readmission was higher in the frail group than in the nonfrail group. Among all readmitted patients, the frail group had a higher number of grade ≥IV patients requiring ICU treatment. The frail group’s 90-day mortality was 1.3%; the difference was statistically significant (nonfrail: 0.3%, *P*=0.021). In the univariate and multivariate logistic regression analyses, mFI ≥0.27 (odds ratio 3.231, 95% CI: 1.889−5.523, *P*<0.001), extended pancreatectomy, BMI ≥30 kg/m^2^, male sex, and malignancy were risk factors for Clavien–Dindo classification grade ≥III.

**Conclusion:**

mFI is a potential preoperative tool for predicting severe postoperative complications, including mortality, in patients who have undergone MIDP for left-sided tumors.

## Introduction

Highlights

- The modified frailty index can help predict severe postoperative complications in patients who have undergone minimally invasive distal pancreatectomy for left-sided tumors.
- Frail patients had higher rates of severe postoperative complications, including 90-day mortality, than nonfrail patients.

The Miami International Evidence-Based Guidelines on Minimally Invasive Pancreas Resection recommend minimally invasive distal pancreatectomy (MIDP) over open distal pancreatectomy (ODP) for benign and low-grade malignant tumors because MIDP is associated with a reduced hospital stay, blood loss, and equivalent complication rates^1^. Furthermore, MIDP for pancreatic ductal adenocarcinoma (PDAC) is a feasible, safe, and oncologically equivalent technique in experienced hands.

The steady growth of the elderly population has led to an increasing demand for acute and long-term healthcare services^2,3^. This trend and the incidence of pancreatic tumors that increase with age have elevated the cases of older adult patients who have undergone MIDP for a left-sided pancreatic tumor^3–5^. Hence, predictors of postoperative outcomes need to be elucidated for older adults undergoing MIDP. Pancreatic surgery outcomes estimated using risk prediction tools rely on patient-related and tumor-related factors; some of the factors cannot be assessed preoperatively^6,7^. Although recent studies have devised models based on parameters measured preoperatively, these models require high-quality imaging and interpretation and have not been validated with large patient samples from the general population^7–9^. Furthermore, cachexia has often been combined with other parameters to evaluate decreased physiologic reserve; however, this approach requires the evaluation of complex imaging parameters^10^. A multifactorial measure of the overall physiologic reserve, such as frailty, may be a more accurate predictor of outcomes after this high-risk procedure.

The physiologic reserve is not always associated with age. Frailty, defined as a progressive physical and mental loss of function and vitality, with or without coexisting diseases^11^, is highly prevalent among older adults. However, chronological age alone is a poor predictor of adverse outcomes after acute stress^7,11,12^. The Canadian Study of Health and Aging has created a standardized frailty index (CSHA-FI) based on a cumulative deficit model^13^. This model defines frailty as the cumulative effect of individual deficits based on clinical signs, symptoms, disease states, and disabilities, providing a more accurate assessment of aging than chronological age^7,14^. The modified frailty index (mFI), the simplified form of the frailty index, accurately predicts postoperative morbidity and mortality after vascular surgery or colectomy and outcomes in other patient populations^7,15–17^. Although the safety and feasibility have been described in previous studies by comparing the postoperative outcomes of MIDP and ODP according to the CSHA-FI or mFI^4,18^, no studies have specifically examined the mFI for MIDP. Therefore, this study aimed to compare the postoperative outcomes of MIDP for left-sided pancreatic tumors based on mFI.

## Material and methods

### Patient selection

**Figure 1:**
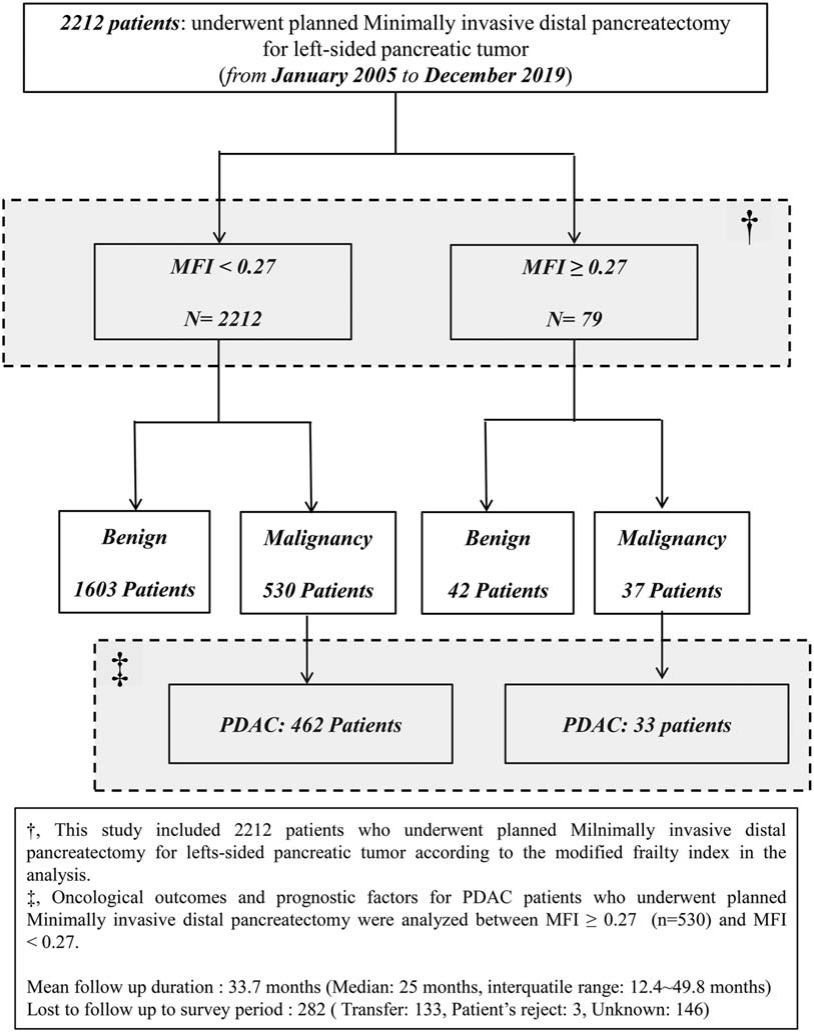
Flowchart of the study. This retrospective cohort study included 2212 patients of MIDP for left-sided pancreatic tumors performed between January 2005 and December 2019. The study population was divided into the frail (*n*=79) and nonfrail (*n*=2133) groups. In addition, a subanalysis of 495 cases of MIDP for PDAC was conducted for the two groups to compare oncological outcomes according to mFI. mFI, modified Frailty Index; MIDP, minimally invasive distal pancreatectomy; PDAC, pancreatic ductal adenocarcinoma.

This retrospective cohort study included 2212 cases of MIDP for left-sided pancreatic tumors performed at our center between January 2005 and December 2019. The study population was divided into the frail (*n*=79) and nonfrail groups (*n*=2133); the postoperative outcomes, including complications, were analyzed according to mFI. In addition, a subanalysis of 495 cases of MIDP for PDAC was conducted for the two groups to compare oncological outcomes according to mFI. Figure 1 describes the study population. Data collection and analysis were performed according to institutional guidelines. This study conformed to the Declaration of Helsinki, and institutional review board approval was obtained accordingly. The study was registered at ClinicalTrials.gov (NCT05837793). This study was described according to the strengthening the reporting of cohort studies in surgery (STROCSS) criteria (Supplemental Digital Content 1, http://links.lww.com/JS9/A920)^19,20^.

### MIDP procedure

As described in a previous study^5^, if there was no invasion to surrounding structures or major blood vessels, minimally invasive surgery was the first to be considered at our hospital for benign and malignant lesions of the neck, body, or tail of the pancreas. MIDP was performed through four ports (two 12 mm ports and two 5 mm or 8 mm ports) with the patients in the supine position^5,21^. The lesser sac was accessed by dividing the gastrocolic ligament. The stomach was retracted upward by a stay suture placed in its posterior wall and pulled outside the abdomen using an Endo Close (Medtronic) device. Subsequently, the spleen was mobilized from the splenic flexure and proximal descending colon. Later, tunneling under the pancreas was performed by dissecting the inferior pancreatic border over the superior mesenteric and splenic veins. The pancreas was transected over 2 min using a linear stapler. The splenic vein was identified along the lower edge of the pancreas, with small branches ligated to separate the pancreas from the vein. The splenic artery was dissected posterior to the vein along the superior border of the pancreas. The vein and artery were transected with multiple clips, followed by antegrade pancreatic division. A medial-to-lateral dissection of the lower pancreatic border was performed.

### Definition of mFI and cutoff value

mFI includes the following 11 items from the National Surgical Quality Improvement Program (NSQIP): diabetes; functional status (not independent); chronic obstructive pulmonary disease (COPD) or pneumonia; congestive heart failure; history of myocardial infarction; hypertension requiring medication; peripheral vascular disease or rest pain; impaired sensorium; history of either transient ischemic attack or cerebrovascular accident; history of cerebrovascular accident with neurologic deficit; and prior percutaneous coronary intervention, previous coronary surgery, or history of angina. Each item was allocated the same weight (1 point) in the calculation of the index^7,22^. mFI was calculated as the proportion of the 11 items used in the study from a given patient (total points as the sum of all the items divided by 11). For example, if there were two deficit items, the mFI would be calculated as 0.18, and if there were three items, the mFI would be calculated as 0.27. Although mFI is not meant to be a dichotomous variable, a cutoff of 0.27 was used based on previous studies^7,23,24^.

### Assessment of postoperative outcomes and data collection

The medical records of the 2212 enrolled patients were reviewed retrospectively. Their clinical, pathological, and surgical data were collected using the electronic medical records of our institute as follows: age at diagnosis, sex, BMI, American Society of Anesthesiologists (ASA) classification score, Charlson comorbidity index score, mFI, operative time, rate of conversion to open surgery, length of hospital stay (LOHS), extended pancreatectomy, 90-day mortality, and pathological outcomes. Clinically relevant postoperative pancreatic fistula (CR-POPF), overall complications, and extended pancreatectomy were assessed and graded based on the criteria of the International Study Group of Pancreatic Surgery and the Clavien–Dindo classification of surgical complications^7,22,25–28^. Major complications were defined as Clavien–Dindo class III or IV. Complications were divided into two categories: those that occurred after surgery and before discharge were classified as ʻcomplications before dischargeʼ, and those that occurred within 90 days after surgery and readmission were classified as ʻcomplications during readmissionʼ. For the subanalysis of MIDP for PDAC, data on neoadjuvant therapy, adjuvant therapy, recurrence, lymphovascular invasion, and perineural invasion were investigated. Tumor, node, and metastasis staging was classified according to the American Joint Committee on Cancer (AJCC) manual, 8th edition^29^. Resection margins were categorized according to the distance between the margin and tumor as R0 (≥1 mm), R1 (<1 mm), or R2 (macroscopically positive)^30^. We considered patients who had a recurrence but did not visit the hospital for greater than 1 year or who did not visit for greater than 1 year within 5 years after surgery as participants lost to follow-up. Death certificates and the time of death were confirmed by a National Health Insurance inquiry. Recurrence was confirmed by reviewing surgical and oncological electronic medical records.

### Statistical analysis

Variables are presented as frequencies, percentages, means with SD, or medians with an interquartile range, depending on their types. Statistical analysis was performed using the Student’s *t*-test for continuous variables and the *χ* ^2^ test for binary outcomes. Univariate and multivariate logistic regression analyses were conducted for factors influencing open conversion and complications with grade ≥3. Survival analysis and the determination of differences between survival estimates were performed using the Kaplan−Meier method with the log-rank test. Univariate and multivariate Cox regression analyses were used for prognostic factors influencing 5-year overall survival after MIDP for PDAC. The threshold for significance was set at *P<*0.05. All statistical analyses were conducted using IBM SPSS version 21.0 (IBM Inc.).

## Results

In a previous study, we stated that the number of older adult patients over 70 years of age increased from 1.0 to 22.1%, and the proportion of octogenarians in the population has also increased over the decade^5^. Frail patients make up an annual average of 3.6 and 1.3−4.9% of the population: 2005 (0%), 2006 (0%), 2007 (4.2%), 2008 (1.7%), 2009 (1.3%), 2010 (4.2%), 2011 (3.6%), 2012 (3.3%), 2013 ( 4.5%), 2014 (4.5%), 2015 (4.9%), 2016 (3.8%), 2017 (3.7%), 2018 (3.3%), and 2019 (2.2%). Of the 2212 patients in this study, 79 (3.6%) belonged to the frail group. Furthermore, among 335 patients over 70 years of age, 43 (12.8%) were frail.

### Demographic features according to mFI

Table 1 summarizes the demographic features of the patients. The number of male patients; those with an age greater than or less than 70 years, ASA score ≥3, Charlson comorbidity index score ≥6, and malignancy; and those who underwent extended pancreatectomy were significantly higher in the frail group than in the nonfrail group. Over 85% of the frail patients had diabetes mellitus, hypertension, a history of peripheral vessel disease, a totally or partially dependent health status, congestive heart failure within 30 days before surgery, myocardial infarction before 6 months, and a history of severe COPD. Previous percutaneous coronary intervention, history of impaired sensorium and transient ischemic attack, and cerebrovascular accident or stroke with neurologic deficit were also common in the frail group.

**Table 1:** Demographic features according to the mFI.

Factors		Overall (*n*=2212)	Nonfrail (mFI <0.27) *n*=2133	Frail (mFI ≥0.27) *n*=79	*P* ^a^
Sex	*n* (%)				<0.001
** **Female		1284 (58.0)	1256 (58.9)	28 (35.4)	
** **Male		928 (42.0)	877 (41.1)	51 (64.6)	
Age (years)	Mean, SD	54.5±14.4	53.9±14.3	68.8±8.4	<0.001
Age ≥70 years	*n* (%)	335 (15.1)	292 (13.7)	43 (54.4)	<0.001
BMI ≥30 kg/m2	*n* (%)	65 (2.9)	62 (2.9)	3 (3.8)	0.503
ASA score ≥3	*n* (%)	112 (5.0)	92 (4.3)	20 (25.3)	<0.001
Charlson comorbidity index score ≥6	*n* (%)	121 (5.5)	88 (4.1)	33 (41.8)	<0.001
Diagnosis	*n* (%)				<0.001
** **Malignancy		567 (25.6)	530 (24.8)	37 (46.8)	
** **PDAC		495 (23.2)	462 (21.7)	33 (41.8)	
** **Others		72 (2.4)	68 (3.1)	4 (5.0)	
** **Benign		1645 (74.4)	1603 (75.2)	42 (53.2)	
Type of the MIDP	*n* (%)				0.436
** **Laparoscopic		2096 (94.8)	2019 (94.7)	77 (97.5)	
** **Robotic		116 (5.2)	114 (5.3)	2 (2.5)	
Conversion to open procedure	*n* (%)	78 (3.5)	70 (3.3)	8 (10.1)	0.006
Extended pancreatectomy	*n* (%)	286 (12.9)	268 (12.6)	18 (22.8)	0.015
Preoperative variables included in the mFI calculation	*n* (%)				
Functional health status before surgery (totally or partially dependent)		18 (0.8%)	4 (0.2%)	14 (17.7%)	<0.001
** **Diabetes mellitus		475 (21.5%)	405 (19.0%)	70 (88.6%)	<0.001
** **Hypertension		637 (28.8%)	562 (26.3%)	75 (94.9%)	<0.001
** **History of PVD		25 (1.1%)	17 (0.8%)	8 (10.1%)	<0.001
** **CHF within 30 days before surgery		4 (0.2%)	1 (0.1%)	3 (3.8%)	<0.001
** **MI 6 months before surgery		4 (0.2%)	1 (0.1%)	3 (3.8%)	<0.001
** **History of severe COPD		23 (1.0%)	13 (0.6%)	10 (12.7%)	<0.001
** **Previous PCI		49 (2.2%)	21 (1.0%)	28 (35.4%)	<0.001
** **History of impaired sensorium		8 (0.4%)	2 (0.1%)	6 (7.6%)	<0.001
** **History of transient ischemic attack		8 (0.4%)	5 (0.2%)	3 (3.8%)	0.002
** **CVA/stroke with neurologic deficit		38 (1.7%)	16 (0.8%)	22 (27.8%)	<0.001

^a^ *P*-values are calculated using the *χ* ^2^ test for binary variables

ASA classification, American Society of Anesthesiologists physical status classification; CHF, congestive heart failure; COPD, chronic obstructive pulmonary disease; CVA, cerebrovascular accident; IQR, interquartile range; mFI, modified frailty index; MI, myocardial infarction; PCI, percutaneous coronary intervention; PDAC, pancreatic ductal adenocarcinoma; PVD, peripheral vascular disease.

### Postoperative outcomes and complications according to mFI

Operative time, readmission, and POPF did not differ between the frail and the nonfrail groups. However, CR-POPF was significantly (*P*=0.003) higher in the frail group (25.3%) than in the nonfrail group (12.5%). Overall complications with grade ≥3 were also significantly higher in the frail group (26.6%) than in the nonfrail group (8.5%). Furthermore, the proportion of all complications before readmission was higher in the frail group than in the nonfrail group (grade ≥III: 4.2 vs. 25.3%, *P*<0.001; grade ≥IV: 0.3 vs. 6.3%, *P*<0.001). Among all readmitted patients, the frail group had a higher number of grade ≥IV patients requiring ICU treatment (0.3 vs. 2.5%, *P*=0.026). The frail group’s 90-day mortality was 1.3%, with a statistically significant difference (*P*=0.021). Table 2, Table S1 (Supplemental Digital Content 2, http://links.lww.com/JS9/A921) and Table S2 (Supplemental Digital Content 3, http://links.lww.com/JS9/A922) summarizes the postoperative outcomes.

**Table 2:** Postoperative outcomes according to the mFI.

Factors		Overall (*n*=2212)	Nonfrail (mFI <0.27) *n*=2133	Frail (mFI ≥0.27) *n*=79	*P* ^a^
Operative time (min)	Mean, SD	190.9±66.0	190.3±65.3	205.1±81.9	0.118
Length of hospital stay (days)	Mean, SD	9.1±6.1	8.8±5.1	15.9±17.1	<0.001
Overall POPF^b^	*n* (%)	878 (39.7)	842 (39.5)	36 (45.6)	0.293
** **CR-POPF		286 (12.9)	266 (12.5)	20 (25.3)	0.003
Complication† grade ≥3, overall	*n* (%)	203 (9.2)	182 (8.5)	21 (26.6)	<0.001
Complication before discharge	*n* (%)				
** **grade ≥III		109 (4.9)	89 (4.2)	20 (25.3)	<0.001
** **grade ≥IV		12 (0.5)	7 (0.3)	5 (6.3)	<0.001
Readmission	*n* (%)	161 (7.3)	152 (7.1)	9 (11.4)	0.181
Complications during readmission	*n* (%)				
** **grade ≥III		113 (5.2)	108 (5.1)	5 (6.3)	0.601
** **grade ≥IV		8 (0.4)	6 (0.3)	2 (2.5)	0.026
90-day mortality	*n* (%)	4 (0.2)	3 (0.1)	1 (1.3)	0.021

^a^ *P*-values are calculated using the *χ* ^2^ test for binary variables.

^b^ Postoperative pancreatic fistula (POPF) and clinically relevant POPF (CR-POPF) and overall complications were assessed and graded based on the criteria of the International Study Group of Pancreatic Fistula and the Clavien–Dindo complication classification, respectively.

mFI, modified frailty index.

### Univariate and multivariate logistic regression analyses of risk factors for Clavien–Dindo classification grade ≥3

Extended pancreatectomy [odds ratio (OR) 1.528, 95% CI: 1.042−2.242, *P*=0.031), BMI ≥30 kg/m^2^ (OR 2.135, 95% CI: 1.076−4.235, *P*=0.031), mFI ≥0.27 (OR 3.231, 95% CI: 1.889−5.523, *P*<0.001), male sex (OR 1.631, 95% CI: 1.206−2.204, *P*=0.001), and malignancy (OR 1.604, 95% CI: 1.143−2.249, *P*=0.006) were risk factors for Clavien–Dindo classification grade ≥3 (Table 3).

**Table 3:** Univariate and multivariate logistic regression analyses based on overall severe complications after MIDP.

	Univariate			Multivariate		
Factors	OR	95% CI	*P*	OR	95% CI	*P* ^a^
Extended pancreatectomy^b^ (yes)	2.113	1.487−3.002	<0.001	1.528	1.042−2.242	0.031
Comorbidity (yes)	1.395	1.045−1.863	0.024			
BMI ≥30 (kg/m2)	2.074	1.067−4.034	0.032	2.135	1.076−4.235	0.031
ASA classification ≥3	1.758	1.028−3.007	0.039			
Modified frailty index ≥0.27	3.881	2.303−6.540	<0.001	3.231	1.889−5.523	<0.001
Charlson comorbidity index score ≥6	1.204	0.665−2.182	0.541			
Sex (male)	1.989	1.493−2.651	<0.001	1.631	1.206−2.204	0.001
Malignancy (yes)	1.942	1.445−2.610	<0.001	1.604	1.143−2.249	0.006
Open conversion (yes)	2.354	1.316−4.213	0.004			
Operative time (min)	1.005	1.003−1.007	<0.001			
Age ≥70 years	0.578	0.138−2.424	0.454			

^a^ *P*-values are calculated using the logistic regression analysis.

^b^ Extended pancreatectomy is defined according to the International Study Group of Pancreatic Fistula.

ASA classification, American Society of Anesthesiologists physical status classification; MIDP, minimally invasive distal pancreatectomy; OR, odds ratio.

### Subanalysis for oncological outcomes of MIDP for PDAC according to mFI

**Figure 2:**
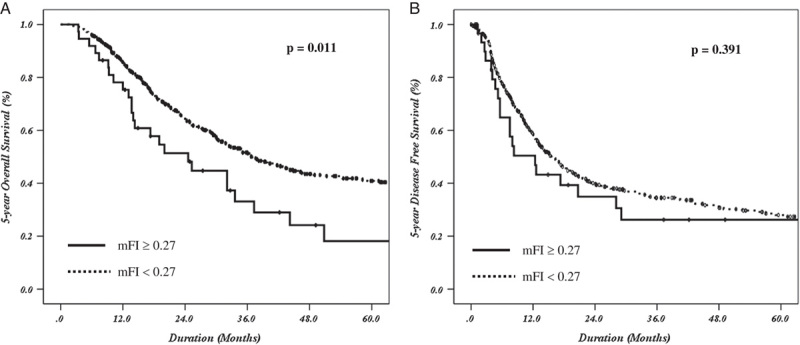
The 5-year overall survival and disease-free survival of the 495 patients with PDAC who underwent MIDP according to mFI. (A) The nonfrail group showed significantly higher 5-year overall survival than the frail group (40.9 vs. 18.1%, *P*=0.011). The median recurrence times in the nonfrail and frail groups were 36.9 months and 24.6 months, respectively. (B) The 5-year disease-free survival did not differ between both groups (28.0 vs. 26.2%, *P*=0.391). The median recurrence times in the nonfrail and frail groups were 15.8 months and 12.4 months, respectively. mFI, modified Frailty Index; MIDP, minimally invasive distal pancreatectomy; PDAC, pancreatic ductal adenocarcinoma.

After the first MIDP for PDAC in 2009, 495 MIDPs were performed by 2019. We compared the 5-year overall survival of the 495 patients with PDAC who underwent MIDP according to mFI (462 nonfrail vs. 33 frail patients) and found a significant between-group difference (40.9 vs. 18.1%, *P*=0.011; Fig. 2A). The median survival times in the nonfrail and frail groups were 36.9 months and 24.6 months, respectively. The 5-year disease-free survival did not differ between the two groups (28.0 vs. 26.2%, *P*=0.391; Fig. 2B). The median recurrence times in the nonfrail and frail groups were 15.8 months and 12.4 months, respectively. When univariate and multivariate Cox regression analyses were performed based on the 5-year overall survival after MIDP for left-sided PDAC, AJCC stage ≥III, lymphovascular invasion, and chemotherapy were identified as prognostic factors (Table 4).

**Table 4:** Univariate and multivariate Cox regression analyses based on the 5-year overall survival after MIDP for left-sided PDAC.

	Univariate			Multivariate		
Factors	HR^a^	95% CI	*P* ^a^	HR	95% CI	*P*
R0 resection^c^ (yes)	2.104	1.632−2.714	<0.001			
AJCC stage^d^ ≥III	2.224	1.689−2.927	<0.001	2.142	1.524−3.011	<0.001
Lymph node ≥N1	1.792	1.398−2.298	<0.001			
Perineural invasion (yes)	1.974	1.415−2.752	<0.001			
Lymphovascular invasion (yes)	1.986	1.553−2.540	<0.001	1.478	1.088−2.007	0.012
Charlson comorbidity index score ≥6	1.320	0.993−1.756	0.056			
Modified frailty index ≥0.27	1.691	1.121−2.551	0.012			
Sex (male)	1.411	1.109−1.795	0.005			
Chemotherapy completion (yes)	0.215	0.159−0.290	<0.001	0.224	0.165−0.305	<0.001
Open conversion (yes)	1.641	1.161−2.318	<0.001			
Age ≥70 years	1.408	1.096−1.810	0.007			

^a^ HR, hazard ratio, estimated using Cox regression models excluding possible confounding variables.

^b^ *P*-values are calculated using the Cox proportional hazards model.

^c^ R0 is a distance of >1 mm from the tumor to the resection margin.

^d^ The T, N, and M stages are based on the American Joint Committee on Cancer (AJCC) manual, 8th edition.

MIDP, minimally invasive distal pancreatectomy; PDAC, pancreatic ductal adenocarcinoma.

## Discussion

The present study showed that frailty is associated with worse outcomes after MIDP. As per previous studies, frailty is a known predictor of postoperative morbidity, mortality, and a longer LOHS^23,31^. Although single-organ assessment should not be ignored, preoperative recognition of factors indicating geriatric vulnerability may provide important insights into predicting postoperative complications and deaths. Traditionally, frailty has been described in the form of physical weakness as a function of aging. However, besides chronological age, several other factors contribute to physiologic aging and determine functional reserve and response to stress^7,10,11^. Surgeons often decide whether or not to operate based on patient age without fully considering the physiological reserve. Patients tend to overestimate their abilities when it comes to outcomes and recovery after major surgery^7,32^.

Several studies have explored the prognostic indicator of mFI in pancreatic surgery. However, our study differs from previous research in several aspects. Mogal *et al*.^7^ reported that high mFI was associated with increased postoperative morbidity (OR 1.68, 95% CI: 1.43–1.97, *P*<0.001) and mortality (OR 2.45, 95% CI: 1.74–3.45, *P*<0.001) in patients undergoing Whipple’s operation. Augustin *et al*.^33^ reported on the prognostic value of mFI, including ODP. However, their study included both Whipple’s operation and a mix of open and minimally invasive procedures, leading to heterogeneity in the patient population compared to our study. The recent study by Paiella *et al*.^4^ focused on examining the postoperative course in elderly individuals aged greater than or equal to 70 years, comparing MIDP and ODP. Their study primarily aimed to evaluate the surgical course based on mFI in older adults. The results showed that the severity of the postoperative course increased with mFI. However, their study included only 42 out of the 204 elderly patients who underwent MIDP.

Other studies, such as the one by Konstantinidis *et al*.^18^, highlighted the differences between MIDP and ODP in frail patients. However, there were limitations in assessing the prognostic value of mFI for MIDP in severely frail patients. In their study, out of 1038 frail patients, only 5 were classified as severely frail, and among the 480 patients who underwent MIDP, only 1 was classified as severely frail. This poses limitations in confirming the prognostic value of mFI, specifically in severely frail patients undergoing MIDP. In our study, we aimed to confirm the role of mFI as a prognostic tool in a larger patient population of 2212 individuals who underwent MIDP across all age groups. According to the Miami guidelines published in 2020, MIDP has become the standard treatment for left-sided benign and low-grade malignant tumors^1^. For PDAC, MIDP appears to be a feasible, safe, and oncologically equivalent technique in experienced hands. Based on these reasons, we aimed to investigate the utility of mFI as a predictive tool that can be assessed preoperatively in patients of all ages who undergo MIDP. To our knowledge, no studies have specifically addressed this research question.

Several studies have reported the safety and feasibility of pancreatectomy and MIDP in older adults^34–36^. However, evaluating the patient’s risks based solely on age is difficult owing to limitations in reflecting physiological age, functional reserve, and responses to the risk of postoperative complications. In the present study, age alone was insufficient in predicting risk factors associated with severe complication rates in patients aged greater than 70 years. Of the 335 patients aged greater than 70 years, 54.4% were in the frail group; 13.7% of patients in this group were below 70 years of age. However, the rate of severe complications after MIDP in patients aged greater than or equal to 70 years was 8.7%, which was not significantly different from that in patients aged less than 70 years (9.3%, *P*=0.837).

Furthermore, no significant difference was observed in CR-POPF, 90-day mortality, and readmission rates based on the age of greater than or equal to 70 years. Age greater than or equal to 70 years was not a significant prognostic factor in the univariate analysis of severe complications (OR 0.578, 95% CI: 0.138−2.424, *P*=0.454; Table 3). When classified based on mFI greater than or equal to 0.27, the severe complication rate was significantly higher in the frail group than in the nonfrail group. In the multivariate analysis, mFI greater than or equal to 0.27 was a significantly poor prognostic factor (Table 3). In patients with complications before discharge (6.3 vs. 0.3%, *P*<0.001) and during readmission (2.5 vs. 0.3%, *P*=0.026), complications of grade ≥IV requiring ICU treatment were significantly higher in the frail group than in the nonfrail group (Table 2). Notably, the frail group’s 90-day mortality was also higher (1.3 vs. 0.1%, *P*=0.021).

Seven patients in the frail group had the following grade ≥IV complications requiring ICU treatment: pseudoaneurysm rupture (two patients), postoperative bleeding, cerebral infarction, aspiration pneumonia with cerebral infarction, postoperative colon perforation, and Stevens−Johnson syndrome; of these patients, the one with Stevens−Johnson syndrome died. When analyzing the relationship between mFI and severe complications using receiver operating characteristic curves, the area under the curve (AUC) for mFI was 0.6483 (95% CI: 0.6091–0.6876). In comparison, the AUC for the Charlson comorbidity index was 0.5532 (95% CI: 0.5125–0.5939), and the AUC for the ASA score was 0.5280 (95% CI: 0.4973–0.5588). The estimated difference in AUC between mFI and the Charlson comorbidity index was 0.0951 (*P*<0.001). The estimated difference in AUC between mFI and the ASA score was 0.1288 (*P*<0.001). The observed statistically significant differences in AUC highlight the enhanced predictive accuracy of mFI for identifying individuals at risk of severe complications.

Existing morbidity evaluation tools require imaging tests or have many items when used in actual clinical practice, making them difficult to use as screening tools. Although recent studies have devised models based on parameters measured preoperatively, they require high-quality imaging and interpretation and have not been validated using large patient samples from the general population^7–9^. The Physiological and Operative Scoring System for enumeration of morbidity and mortality (POSSUM) and the Estimation of Physiologic Ability and Surgical Stress (E-PASS) are complex and have shown inconsistent results in predicting morbidity and mortality in patients undergoing pancreatic surgery^37–39^. Sarcopenia has also been combined with other parameters for assessing decreased physiologic reserve. However, this approach requires the evaluation of complex imaging parameters^10^. A multifactorial measure of the overall physiologic reserve, such as frailty, may be a more accurate predictor of outcomes after MIDP. The mFI can be used to collect information reliably and consistently before surgery. Furthermore, it is considered a readily available frailty-assessment tool that uses simple historical parameters. The mFI includes the following 11 items from the NSQIP: diabetes; functional status (not independent); COPD or pneumonia; congestive heart failure; history of myocardial infarction; hypertension requiring medication; peripheral vascular disease or rest pain; impaired sensorium; history of either a transient ischemic attack or cerebrovascular accident; history of a cerebrovascular accident with neurologic deficit; and prior percutaneous coronary intervention, previous coronary surgery, or history of angina. Each item was allocated the same weight (1 point) in the calculation of the index^7,22^.

The subanalysis of patients who underwent MIDP for PDAC showed a poor 5-year overall survival in the frail group (Fig. 2A). However, in the multivariate analysis, mFI was not included as a survival-related prognostic factor (Table 4). According to our data, the survival differences (*P*=0.049) were 32.7% (chemotherapy completion) and 9.4% (no chemotherapy completion), and the median times were 31.9 months and 14.3 months, respectively. When chemotherapy was completed, no difference was observed in the 5-year overall survival between the nonfrail (38.0%) and frail (32.8%) groups (*P*=0.661, median time 34.8 months and 33.6 months, repectively). The chemotherapy rate was lower in the frail group (63.6%) than in the nonfrail group (74.9%); however, this difference was not statistically significant (*P*=0.155). Similarly, the chemotherapy completion rate was lower in the frail group (65.0%) than in the nonfrail group (74.6%), and the difference was not statistically significant (*P*=0.431). These findings suggest that frailty may negatively affect the implementation and completion of chemotherapy; however, this requires validation. Hence, further large-scale analysis is required in the future to confirm and clarify such findings.

This study had several limitations. First, we selected 11 of the 70 variables described in the original CSHA mapped to the preoperative variables employed in the NSQIP dataset. These selected variables may not fully represent the full spectrum of aging parameters. Second, the variables used in mFI are based on subjective evaluation. The NSQIP database is also not specifically designed to assess vulnerabilities. Therefore, its use for preoperative patient evaluation needs to be validated in prospective studies. Third, since this was a retrospective study, a selection bias could have possibly targeted patients with relatively few underlying diseases who could be operated on. Also, the populations of the frail and nonfrail groups were not homogeneous due to the unequal group sizes, which should be considered when interpreting the study findings. Finally, this was a single-center study, and the results may not be generalizable to other institutions.

Nonetheless, our study has several important implications. First, the variables used to calculate mFI were objective, readily available from history, and easily reproducible. Identifying frailty allows an objective assessment of the onset of complications and the likelihood of recovery. This, in turn, could help better inform the shared decision-making process essential for preoperative consultation and patient selection. Second, frailty is not an irreversible state; rather, there is a transition between higher and lower states of frailty. Therefore, implementing prerehabilitation strategies to identify frail patients and address modifiable frailty-related factors can prevent worsening physical and functional impairments and potentially improve postoperative outcomes. For patients who can be easily screened for frailty using mFI, multidisciplinary care, including geriatric clinics, is expected to help mitigate risks associated with specific preoperative conditions.

## Conclusion

The mFI is a potential preoperative tool for predicting severe postoperative complications in patients who have undergone MIDP. It can help reduce postoperative risk through multidisciplinary care while screening patients with frailty preoperatively. Further prospective studies are necessary to confirm the value of mFI in predicting high-risk groups preoperatively to manage modifiable frailty-related factors, thereby reducing postoperative complications.

## Ethical approval

This study conformed to the Declaration of Helsinki, and institutional review board of Asan medical center approval was obtained accordingly. Approval number was 2022-1709.

## Consent

This study did not involve personal privacy and commercial interests and collected patient diagnosis and treatment information retrospectively from the medical system of our hospital, which was almost no risk to patients. After reviewed by the Ethical Committee of Asan medical center, our study was approved exemption from signing informed consent.

## Sources of funding

This study was conducted without any involvement or funding from sponsors.

## Author contribution

Y.P., D.W.H., J.H.L., K.B.S., W.L., B.J.K., E.S.J., and S.C.K.: acquisition of data; Y.P., D.W.H., J.H.L., K.B.S., W.L., B.J.K., E.S.J., and S.C.K.: analysis and interpretation of data; Y.P. and S.C.K.: statistical analysis; Y.P.: drafting of manuscript; Y.P.: critical revision; Y.P. and S.C.K.: thereafter, in the final revising process of this manuscript. All authors discussed the interpretation of the data and intellectual contents.

## Conflicts of interest disclosure

The authors declare that they have no conflicts of interest.

## Research registration unique identifying number (UIN)

1. Name of the registry: ClinicalTrials.gov.
2. Unique identifying number or registration ID: NCT05837793.
3. Hyperlink to your specific registration (must be publicly accessible and will be checked): https://classic.clinicaltrials.gov/ct2/show/NCT05837793?type=Obsr&cntry=KR&draw=5&ran k=31.

## Guarantor

Correspondence to: Song Cheol Kim, MD, PhD, Department of Surgery, Division of Hepatobiliary and Pancreatic Surgery, University of Ulsan College of Medicine, Asan Medical Center, 88 Olympic-ro 43-gil, Songpa-gu, Seoul, 05505, Republic of Korea. Tel.: +82 2 3010 3936, fax: +82 2 474 9027. E**-**mail: drksc@amc.seoul.kr.

## Provenance and peer review

Not applicable.

## Data availability statement

If necessary, the data will be made available for sharing even after the publication of the paper.

Data source: This retrospective cohort study included 2212 cases of MIDP for left-sided pancreatic tumors performed at the Asan Medical Center (Seoul, South Korea) between January 2005 and December 2019.

Data collection method: The medical records of the 2212 enrolled patients were reviewed retrospectively, and their clinical, pathologic, and surgical data were collected using the electronic medical records of our institute. Data collection and analysis were performed according to institutional guidelines. This study conformed to the Declaration of Helsinki, and institutional review board approval was obtained accordingly.

Data cleaning method: Variables are presented as frequencies, percentages, mean with SD, or median with interquartile range, depending on their types. Statistical analysis was performed using Student’s *t*-test for continuous variables and the *χ* ^2^ test for binary outcomes. Univariable and multivariable logistic regression analyses were conducted for factors influencing open conversion and complication ≥grade 3. Survival analysis and the determination of differences between survival estimates were performed using the Kaplan−Meier method with the log-rank test. Univariable and multivariable Cox regression analyses were used for prognostic factors influencing 5-year overall survival after MIDP for PDAC. The threshold for significance was set at *P<*0.05. All statistical analyses were conducted using IBM SPSS version 21.0 (IBM SPSS).

Variables and their definitions: The medical records of the 2212 enrolled patients were reviewed retrospectively, and their clinical, pathologic, and surgical data were collected using the electronic medical records of our institute as follows: age at diagnosis, sex, BMI, American Society of Anesthesiologists (ASA) classification score, Charlson comorbidity index score, mFI, operative time, rate of conversion to open surgery, LOHS, extended pancreatectomy, 90-day mortality, and pathologic outcomes. Clinically relevant postoperative pancreatic fistula (CR-POPF), overall complications, and extended pancreatectomy were assessed and graded based on the criteria of the International Study Group of Pancreatic Surgery and the Clavien–Dindo classification of surgical complications.^7,20,23-26^ Major complications were defined as Clavien–Dindo class III or IV complications. Complications were divided into two categories: Complications that occurred after surgery and before discharge were classified as complications before discharge, and complications that occurred within 90 days after surgery and readmission were classified as complications during readmission. For the subanalysis of MIDP for PDAC, data on neoadjuvant therapy, adjuvant therapy, recurrence, lymphovascular invasion, and perineural invasion were investigated. Tumor, node, and metastasis staging was classified according to the American Joint Committee on Cancer (AJCC) manual, 8th edition.^27^ Resection margins were categorized according to the distance between the margin and tumor as R0 (≥1 mm), R1 (<1 mm), or R2 (macroscopically positive).^28^ We considered patients who had a recurrence but did not visit the hospital for greater than 1 year or who did not visit for greater than 1 year within 5 years after surgery as participants lost to follow-up. Death certificates and the time of death were confirmed by a National Health Insurance inquiry. Recurrence was confirmed by reviewing surgery and oncology EMRs.

## Supplementary Material
