# Supplementary material for: Evaluation of postoperative outcomes of minimally invasive distal pancreatectomy for left-sided pancreatic tumors based on the modified frailty index: a retrospective cohort study
Source: Int J Surg. 2023 Aug 17;109(11):3497–505. doi: 10.1097/JS9.0000000000000670 (PMC10651302; doi:10.1097/JS9.0000000000000670)
Supplement: Supplementary file 2 [file js9-109-3497-s002.docx]

**Table S1. Complications of the patients who underwent minimal invasive distal pancreatectomy before discharge**

| Grade of Complication^*^ | Non-frail (mFI <0.27)  n = 2133 | Frail (mFI ≥0.27)  n = 79 |
| --- | --- | --- |
| No | 1307 (61.3%) | 48 (60.8%) |
| Grade I | 561 (26.3%) | 18 (23.1%) |
| Biochemical leakage | 540 | 14 |
| Chyle leak with low long-chain triglyceride diet | 6 | 2 |
| Ileus | 4 | 2 |
| Intraabdominal fluid collection with conservative care | 2 | 0 |
| Cholangitis with conservative care | 2 | 0 |
| Wound dehiscence with bedside care | 2 | 0 |
| Superficial surgical site infection with bedside care | 1 | 0 |
| Portal vein thrombus with conservative care | 1 | 0 |
| Pulmonary complication | 1 | 0 |
| Others | 2 | 0 |
| Grade II | 176 (8.3%) | 10 (12.8%) |
| POPF grade B with antibiotic therapy | 143 | 5 |
| Portal vein thrombus with anticoagulation therapy | 8 | 0 |
| Intraabdominal fluid collection with antibiotic therapy | 5 | 2 |
| Ileus with total parenteral nutrition therapy | 7 | 0 |
| Superficial surgical site infection with antibiotic therapy | 3 | 1 |
| Post-pancreatectomy hemorrhage (PPH) with transfusion | 2 | 0 |
| Hematoma with antibiotic therapy | 1 | 0 |
| Cholangitis with antibiotic therapy | 1 | 0 |
| Cellulitis with antibiotic therapy | 0 | 1 |
| Pulmonary complication with antibiotic therapy | 1 | 0 |
| Others | 5 | 1 |
| Grade IIIa | 73 (3.4%) | 14 (17.7%) |
| POPF grade B with drainage | 62 | 12 |
| PV thrombus with interventional therapy | 3 | 1 |
| PPH with embolization or stent insertion | 2 | 0 |
| Cholangitis with interventional therapy | 2 | 0 |
| Hematoma with transfusion with drainage | 1 | 0 |
| Others | 0 | 1 |
| Grade IIIb | 9 (0.4%) | 1 (1.2%) |
| Postoperative bleeding with reoperation | 6 | 0 |
| Wound dehiscence with reoperation | 1 | 1 |
| POPF grade C with reoperation | 1 | 0 |
| Others | 1 | 0 |
| Grade IV | 6 (0.3%) | 5 (6.3%) |
| PPH with Intensive Care Unit (ICU) care | 4 | 2 |
| Pulmonary complication with ICU care | 1 | 1 |
| Others | 1 | 2 |
| Grade V | 1 (0.1%) | 0 (0.0%) |
| Postoperative bleeding with sepsis | 1 | 0 |

*, Postoperative pancreatic fistula (POPF) and clinically relevant POPF (CR-POPF) and overall complications were assessed and graded based on the criteria of the International Study Group of Pancreatic Fistula and the Clavien–Dindo complication classification, respectively.
