# Supplementary material for: Evaluation of postoperative outcomes of minimally invasive distal pancreatectomy for left-sided pancreatic tumors based on the modified frailty index: a retrospective cohort study
Source: Int J Surg. 2023 Aug 17;109(11):3497–505. doi: 10.1097/JS9.0000000000000670 (PMC10651302; doi:10.1097/JS9.0000000000000670)
Supplement: Supplementary file 3 [file js9-109-3497-s003.docx]

**Table S2.** Complications of the patients who underwent minimal invasive distal pancreatectomy during readmission

| Grade of Complication^*^ | Non-frail (mFI < 0.27)  n=2133 | Frail (mFI ≥ 0.27)  n=79 |
| --- | --- | --- |
| No | 152 (7.1%) | 9 (11.4%) |
| Grade I | 11 (0.5%) | 0 (0.0%) |
| Ileus | 2 | 0 |
| Intraabdominal fluid collection with conservative care | 1 | 0 |
| Superficial surgical site infection with bedside care | 1 | 0 |
| Portal vein thrombus with conservative care | 3 | 0 |
| Incisional hernia with conservative care | 2 | 0 |
| Others | 2 | 0 |
| Grade II | 35 (1.6%) | 4 (5.1%) |
| POPF grade B with antibiotic therapy | 14 | 1 |
| Portal vein thrombus with anticoagulation therapy | 1 | 0 |
| Intraabdominal fluid collection with antibiotic therapy | 6 | 1 |
| Superficial surgical site infection with antibiotic therapy | 2 | 0 |
| Post-pancreatectomy hemorrhage (PPH) with transfusion | 1 | 0 |
| Hematoma with antibiotic therapy | 1 | 0 |
| Cellulitis with antibiotic therapy | 2 | 0 |
| Pancreatitis | 2 | 0 |
| Others | 6 | 0 |
| Grade IIIa | 82 (3.8%) | 2 (2.6%) |
| POPF grade B with drainage | 42 | 1 |
| PV thrombus with interventional therapy | 1 | 0 |
| PPH with embolization or stent insertion | 5 | 0 |
| Hematoma with transfusion with drainage | 2 | 0 |
| Intraabdominal fluid collection with drainage | 22 | 1 |
| Deep surgical site infection with interventional therapy | 1 | 0 |
| Others | 2 | 0 |
| Grade IIIb | 27 (1.2%) | 1 (1.3%) |
| Postoperative bleeding with reoperation | 1 | 0 |
| Others | 2 | 0 |
| Ileus with reoperation | 8 | 1 |
| Incisional hernia with reoperation | 8 | 0 |
| Port site recurrence with reoperation | 7 | 0 |
| Deep surgical site infection with reoperation | 1 | 0 |
| Grade IV | 4 (0.2%) | 1 (1.3%) |
| PPH with Intensive Care Unit (ICU) care | 3 | 1 |
| Ileus with ICU care | 1 | 0 |
| Grade V | 2 (0.1%) | 1 (1.3%) |
| Postoperative bleeding with sepsis | 1 | 0 |
| Ileus with sepsis | 1 | 0 |
| Steven-Johnson syndrome with sepsis | 0 | 1 |

*, Postoperative pancreatic fistula (POPF) and clinically relevant POPF (CR-POPF) and overall complications were assessed and graded on the basis of the criteria of the International Study Group of Pancreatic Fistula and the Clavien–Dindo complication classification, respectively.
